# Glycan Profiles of gp120 Protein Vaccines from Four Major HIV-1 Subtypes Produced from Different Host Cell Lines under Non-GMP or GMP Conditions

**DOI:** 10.1128/JVI.01968-19

**Published:** 2020-03-17

**Authors:** Shixia Wang, Yegor Voronin, Peng Zhao, Mayumi Ishihara, Nickita Mehta, Mindy Porterfield, Yuxin Chen, Christopher Bartley, Guangnan Hu, Dong Han, Lance Wells, Michael Tiemeyer, Shan Lu

**Affiliations:** a Department of Medicine, University of Massachusetts Medical School, Worcester, Massachusetts, USA; b Worcester HIV Vaccine, Inc., Worcester, Massachusetts, USA; c Complex Carbohydrate Research Center, University of Georgia, Athens, Georgia, USA; d Waisman Biomanufacturing, Madison, Wisconsin, USA; Emory University

**Keywords:** HIV-1, gp120, Env protein, glycosylation, vaccine, HIV-1

## Abstract

Envelope (Env) glycoprotein of human immunodeficiency virus type 1 (HIV-1) is an important target for the development of an HIV vaccine. Extensive glycosylation of Env is an important feature that both protects the virus from antibody responses and serves as a target for some highly potent broadly neutralizing antibodies. Therefore, analysis of glycans on recombinant Env proteins is highly significant. Here, we present glycosylation profiles of recombinant gp120 proteins from four major clades of HIV-1 (A, B, C, and AE), produced either as research-grade material in 293 and CHO cells or as two independent lots of clinical material under good manufacturing practice (GMP) conditions. Almost all potential *N*-linked glycosylation sites were at least partially occupied in all proteins. The occupancy rates were largely consistent among proteins produced under different conditions, although a few sites showed substantial variability even between the two GMP lots. Our data confirmed previous studies in the field, showing an abundance of oligomannose on Env protein, with 40 to 50% of glycans being Man_5_ to Man_9_ on all four proteins under all production conditions. Overall, the differences in occupancy and glycan forms among different Env subtypes produced under different conditions were less dramatic than anticipated, and antigenicity analysis with a panel of six monoclonal antibodies, including antibodies that recognize glycan forms, showed that all four gp120s maintained their antibody-binding profiles. Such findings have major implications for the final production of a clinical HIV vaccine with Env glycoprotein components.

**IMPORTANCE** HIV-1 Env protein is a major target for the development of an HIV-1 vaccine. Env is covered with a large number of sugar-based glycan forms; about 50% of the Env molecular weight is composed of glycans. Glycan analysis of recombinant Env is important for understanding its roles in viral pathogenesis and immune responses. The current report presents the first extensive comparison of glycosylation patterns of recombinant gp120 proteins from four major clades of HIV-1 produced in two different cell lines, grown either under laboratory conditions or at 50-liter GMP scale in different lots. Information learned in this study is valuable for the further design and production of HIV-1 Env proteins as the critical components of HIV-1 vaccine formulations.

## Abstract (precis)

HIV-1 Env protein is a major target for the development of an HIV-1 vaccine. Env is covered with a large number of sugar-based glycan forms; about 50% of the Env molecular weight is composed of glycans. Glycan analysis of recombinant Env is important for understanding its roles in viral pathogenesis and immune responses. The current report presents the first extensive comparison of glycosylation patterns of recombinant gp120 proteins from four major clades of HIV-1 produced in two different cell lines, grown either under laboratory conditions or at 50-liter GMP scale in different lots. Information learned in this study is valuable for the further design and production of HIV-1 Env proteins as the critical components of HIV-1 vaccine formulations.

## INTRODUCTION

The envelope (Env) glycoprotein of human immunodeficiency virus type 1 (HIV-1) plays a key role in viral entry and serves as a major target for a preventive HIV-1 vaccine. Env is heavily glycosylated, with *N*-glycans representing about one-half of its molecular weight. In the course of HIV-1 evolution, either within an individual patient or on the population level, viral gene mutations may lead to the disappearance of certain glycosylation sites and to the appearance of new ones. This shifting glycan shield protects the Env proteins from the engagement of antibodies elicited during the course of viral infection, contributing to the growth of escaping viral mutants in chronic infection (1). Nevertheless, some glycan features are highly conserved even across clades, such that glycans contribute to several key antigenic domains recognized by broadly neutralizing antibodies. For example, the highly conserved N332 glycan is important for the binding of monoclonal antibodies (MAbs) PGT128 and 10-1074, while N160 glycan is recognized by MAbs PG9 and PG16 (2, 3). Another conserved feature of the HIV glycan shield is the abundance of unusual oligomannose forms, which normally serve as intermediates in mammalian glycan synthesis (4–6).

Glycosylation patterns can be expected to vary depending on multiple factors that affect glycoprotein synthesis, including the viral strain, the form in which Env is expressed (gp120, gp140, or gp160), the cell type, the protein expression levels, and even the metabolic state of the cell (7). In past studies, the exact proportions of oligomannose on Env varied from 17% to 98%, with levels of 40 to 75% being common for both monomeric gp120 and native trimers (5, 8, 9). However, analysis of two batches of membrane-anchored Env showed remarkable consistency of forms found at each glycosylation site (9, 10), indicating that glycosylation patterns are generally preserved when the same Env protein is produced under identical conditions and that differences in oligomannose contents reflect either virus- or host cell-specific factors.

In previous studies, viral clade-specific differences in the abundance of oligomannose have been attributed to differences in the total number and regional density of glycosylation sites, with higher glycan densities correlating with greater oligomannose contents (4). However, comparisons of glycosylation of the same Env proteins expressed in 293 and CHO cells revealed mostly similar oligomannose contents, similar occupancies, and similar glycan profiles, with some notable exceptions (11, 12). It was observed that more complex glycans were present on CHO-derived clade C gp120, compared to 293-derived protein, particularly at two sites (N386 and N392).

The increasing understanding of the impact of glycans on HIV Env immunogenicity and the increased focus on recombinant Env proteins after the RV144 trial led to the growing appreciation of the importance of these features for the design of Env-based protein vaccines against HIV-1 (13). In particular, characterization of glycan profiles of recombinant Env proteins will be important for interpreting the resulting antibody responses.

Various approaches for producing and purifying recombinant Env proteins for laboratory research and for clinical studies can be employed, but there is limited information based on well-controlled studies regarding how different approaches may affect Env glycosylation. While transiently transfected 293 cells are often used to produce research-grade proteins, the proteins for clinical use are usually produced in stably transfected CHO cells. Clinical material is usually produced in bioreactors that have larger volumes and reach higher cell densities than those used in research-grade protein production. Diverse purification processes, such as antibody-based affinity columns, size exclusion chromatography, lectin-based columns, or industry-preferred ion-exchange columns, are used both in the laboratory and during clinical-grade protein purification. Recently, a few recombinant good manufacturing practice (GMP)-grade Env-based vaccines have been characterized by analysis of glycans (14, 15); however, the number of Env proteins included in those studies was limited, and the studies did not provide direct comparisons between different cell lines or between GMP and non-GMP production of the same Env proteins.

Here, we expanded the study to characterize the glycosylation profiles of four recombinant gp120 proteins, from four major clades of HIV-1 (subtypes A, B, C, and AE), produced under GMP conditions for a phase I human clinical trial (HIV Vaccine Trials Network [HVTN], protocol 124 [HVTN124]). We analyze two separate GMP lots of the same four gp120 proteins, comparing them to the same four gp120 proteins produced under non-GMP conditions in CHO and 293F cells. Our results provide much-needed information on the Env glycan patterns among different viral clades and between different preparations of the same protein. Such information not only is valuable for better understanding of the variation of Env glycan patterns but also is critical for the establishment of quality control standards for the production of clinical grade Env-based HIV-1 vaccines.

## RESULTS

### HIV-1 gp120 Env proteins from four clades, produced under different conditions.

**FIG 1:**
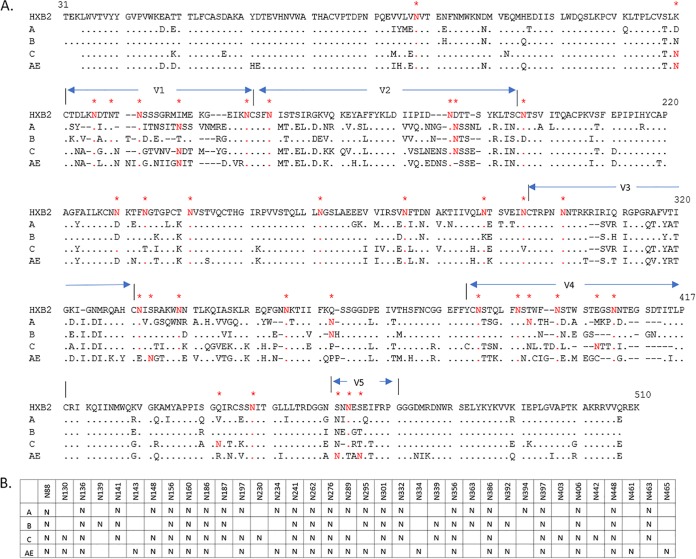
(A) Sequences of the gp120 proteins belonging to clade A (92UG037.8), clade B (JRFL), clade C (93MW965.26), and clade AE (consensus), aligned to the reference strain HXB2. Identical amino acids are shown as dots, gaps are indicated with dashes, and numbers correspond to the HXB2 sequence. Variable regions V1 to V5 of gp120 are indicated above the sequences. PNGSs predicted on the basis of the consensus glycosylation sequence are shown in red and marked with stars above the sequences. (B) Summary of glycosylation site distributions among the four gp120 proteins. N indicates the presence of a PNGS in the sequence.

The four gp120 glycoproteins included in the current study were selected on the basis of the immunogenicity analysis of a large panel of HIV-1 Env variants (16) and were included in a polyvalent DNA prime-protein boost HIV vaccine formulation currently going through a phase I clinical study at HVTN (HVTN124). The glycoproteins represent three primary isolates from clades A, B, and C, as well as a consensus variant from the AE clade. Their amino acid sequences have low homology to each other, in the range of 75 to 80% (Fig. 1A). They have 23 to 26 potential *N*-linked glycosylation sites (PNGSs), which are distributed throughout the sequence in similar but distinct manners (Fig. 1B).

Research-grade gp120 proteins were produced by transient transfection of 293F cells and from stably transfected CHO cells in a laboratory setting, and the proteins were purified using lectin-based columns. For the GMP manufacturing process, stably transfected CHO cells expressing each of the proteins were grown in 50-liter bioreactors, and the purification process involved ion-exchange columns. Two separate GMP manufacturing runs were performed under identical conditions, which allowed us to compare the consistency of glycosylation profiles from one GMP lot to another.

### Glycan analysis of research-grade gp120 proteins.

**FIG 2:**
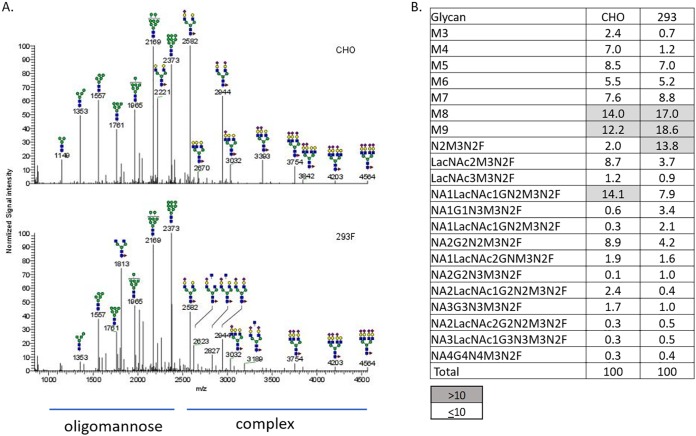
(A) Representative MS spectra of glycans identified on research-grade gp120 proteins produced in CHO cells (top) and 293F cells (bottom). The gp120 protein from clade B (strain JR-FL) is shown here as a representative example. Glycan forms corresponding to the most abundant peaks are shown using the standard glycan icons. MS peaks corresponding to oligomannose and complex glycan forms are indicated. (B) Quantitative comparison of relative proportions of various glycan forms (in percent) on CHO- and 293F-derived gp120-B proteins. Glycan structures found at levels greater than 10% of total glycans are highlighted in gray.

Glycan heterogeneity for gp120 proteins produced as research-grade reagents in the CHO and 293F cell lines was first analyzed. Digestion with peptide-*N*-glycosidase F (PNGase F) was used to release glycans from the gp120 proteins, and the released glycans were permethylated and analyzed by nanospray ionization-multidimensional mass spectrometry (NSI-MSn) to characterize glycan structural features. Representative profiles for clade B gp120 are shown in Fig. 2A. The types of glycan forms found on proteins produced in CHO and 293F cells were generally very similar, and only a few types represented more than 10% of the total glycans (Fig. 2B). Large proportions of oligomannose forms (Man_7_ to Man_9_) were detected in both preparations. A diverse group of complex glycans were also present, as well as some hybrid forms. The results for proteins from three other clades were similar (data not shown).

To study the occupancy rate at each PNGS, proteins were digested with several proteases to produce peptides for liquid chromatography-mass spectrometry (LC-MS) analysis and then were consecutively digested with endo-β-*N*-acetylglucosaminidase H (endo H) and PNGase F to allow detection of occupancy by different types of glycans at each PNGS. Digestion with endo H cleaves *N*-linked glycans between the two *N*-acetylglucosamine (GlcNAc) residues in the core region of the glycan chain on high-mannose and hybrid glycans but not complex glycans, leaving one GlcNAc still bound to the protein. Treatment with PNGase F removes all glycans that have not been affected by endo H treatment and leaves an aspartic acid residue at the site of *N*-linked glycosylation, which can be distinguished from the original asparagine by MS analysis of the peptides. Therefore, consecutive digestion with endo H and PNGase F allowed us to distinguish between oligomannose and complex glycans at each site. The presence of the original asparagine in the peptide indicates that the PNGS has not been glycosylated.

**FIG 3:**
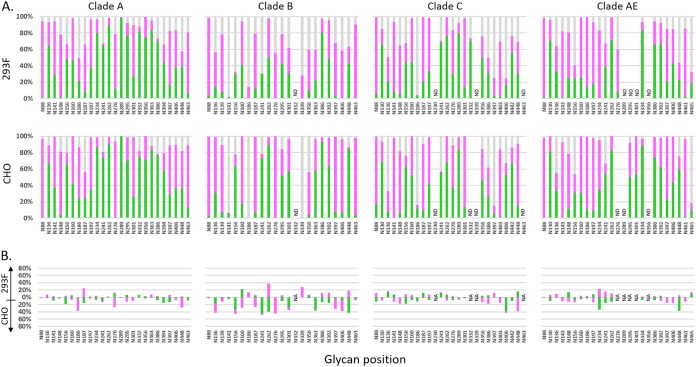
(A) PNGS occupancy analysis of gp120 proteins expressed in 293F cells (top row) and CHO cells (bottom row). The levels of glycan occupancy at each PNGS are shown for each of the gp120 proteins (clade A, B, C, or AE), as indicated above each panel. Green and purple bars indicate the proportions of oligomannose glycans and complex glycans, respectively, and gray bars indicate that the site was not occupied by a glycan. ND indicates that peptides were not detected. (B) Differences in glycan occupancy between the 293F- and CHO-produced proteins at each PNGS for each of the gp120 proteins. Percentages of oligomannose and complex glycans were compared at each site, and the differences were plotted based on which protein had larger amounts of that glycan. For example, at position N187 in clade A protein, the 293F-derived protein had 25 percentage points more complex glycans than the CHO-derived protein, while the CHO-derived protein had 18 percentage points more oligomannose glycans than the 293F-derived protein.

Analysis of the PNGS occupancy of research-grade 293F- and CHO-produced proteins showed that most PNGSs were at least partially occupied in both cases (Fig. 3A). N141, N186, and N339 in clade B proteins, N186 and N397 in clade C proteins, and N465 in clade AE proteins were the only sites that showed less than 20% occupancy in our analysis. Large proportions of oligomannose glycans were observed for all four gp120 proteins, with larger proportions in glycans described as the intrinsic mannose patch.

Glycosylation profiles of proteins produced in 293F and CHO cells were remarkably similar (Fig. 3B). Most of the variation in occupancy was less than 30 percentage points. Clade B proteins showed the largest variation, with the CHO-produced protein being more glycosylated than the 293-produced protein. In some cases, the changes were not in the total occupancy but in the relative abundance of oligomannose and complex glycans. For example, N262 in clade B proteins was almost 100% occupied but the 293-produced protein carried an equal mixture of oligomannose and complex glycans and the CHO-produced protein had almost exclusively oligomannose glycans at this site.

### Glycan analysis of GMP-grade gp120 proteins.

**FIG 4:**
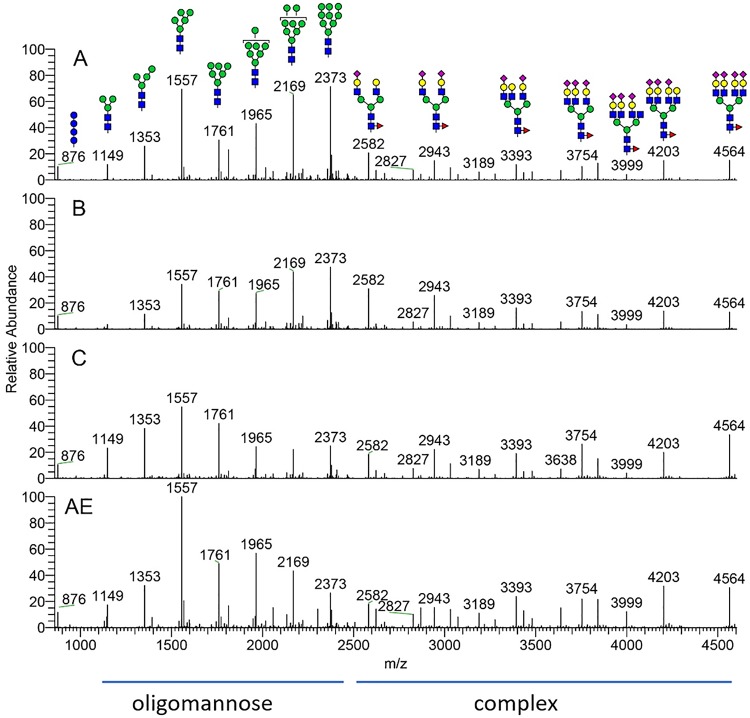
MS spectra of glycans released from four gp120 proteins (clades A, B, C, and AE) produced under GMP conditions. Glycan forms corresponding to the most abundant peaks are shown, using the standard glycan icons. MS peaks corresponding to oligomannose and complex glycan forms are indicated.

**FIG 5:**
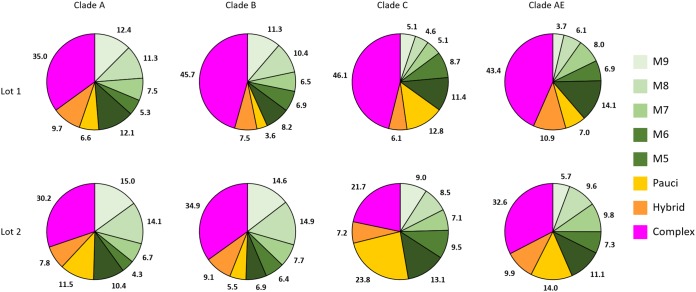
Quantification of the types of glycan forms released from two independent lots (lot 1 and lot 2) of gp120 proteins (clades A, B, C, and AE) produced under GMP conditions. Numbers indicate the percentages of the corresponding glycan forms or structural classes, color coded as Man_5_ (M5), Man_6_ (M6), Man_7_ (M7), Man_8_ (M8), Man_9_ (M9), paucimannose (Pauci) (Man_3_ and Man_4_), hybrid, or complex.

**FIG 6:**
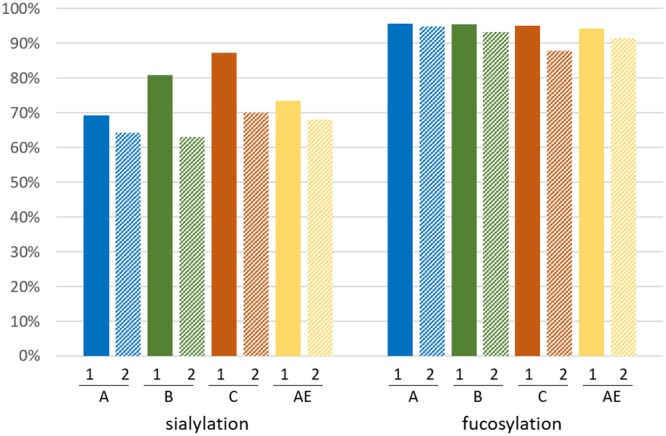
Analysis of sialylation (left) and core fucosylation (right) modifications of complex glycans of each gp120 protein (clade A, B, C, or AE) produced in two different GMP lots. Only complex glycans were included in the analysis. 1 and 2 under the bars of the graph indicate lot 1 and lot 2 GMP-grade gp120 proteins, respectively. A, B, C, and AE indicate gp120 proteins from clades A, B, C, and AE, respectively.

The four GMP-grade gp120 proteins were produced in stably expressing CHO cell lines on a 50-liter scale and were purified using multistep chromatography. Two separate lots were manufactured using the same master cell bank CHO cells and the same fermentation and downstream purification processes, which allowed us to investigate the lot-to-lot variability of GMP-grade gp120 protein preparations. The glycan forms identified for GMP-grade gp120 proteins are shown in Fig. 4, and the relative amounts of these glycan forms in the two GMP lots are show in Fig. 5. For most gp120 proteins, oligomannose (Man_5_ to Man_9_) constituted 40 to 50% of the total glycans, while complex glycans were about 35 to 45% and the rest were hybrid glycans or paucimannose (Man_3_ to Man_4_). The least-processed Man_9_ and Man_8_ forms predominated on clade A and clade B gp120 proteins, while clade C and clade AE proteins showed greater proportions of Man_5_. The proportions of paucimannose showed the greatest variation both between the clades and especially between the two lots of clade C gp120 protein; in one of the lots, paucimannose represented 23% of all glycans. Other proteins showed more consistent glycan compositions in the two independent lots. Complex glycans were predominantly (63 to 87%) sialylated, and >85% were core fucosylated (Fig. 6).

Thus, all variants of GMP-grade gp120 proteins exhibited large proportions of oligomannose glycans. Comparison of the two GMP lots showed mostly comparable glycan compositions for three tested variants of gp120 proteins (clades A, B, and AE) and some variation in the amounts of paucimannose for the clade C variant.

### PNGS occupancy analysis of GMP-grade gp120 proteins.

**FIG 7:**
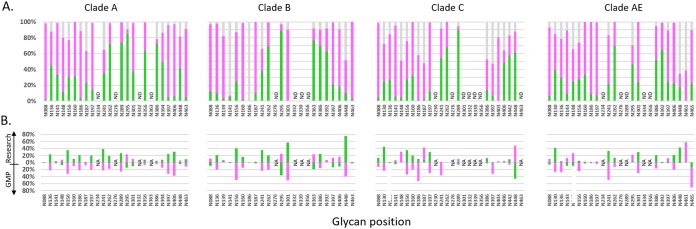
(A) PNGS occupancy analysis of GMP-grade gp120 proteins. The levels of glycan occupancy at each PNGS are shown for each of the gp120 proteins (clade A, B, C, or AE), as indicated above each panel. Green and purple bars indicate the proportions of oligomannose glycans and complex glycans, respectively, and gray bars indicate that the site was not occupied by a glycan. ND indicates that peptides were not detected. (B) Differences in glycan occupancy in gp120 proteins produced in CHO cells under research-grade conditions and under GMP conditions at each PNGS. The plots were generated as described for Fig. 3B.

Next, PNGS occupancy was mapped for proteins from one of the GMP lots and compared to findings for the CHO-produced research-grade proteins. The overall occupancy profiles of the GMP-grade gp120 proteins were generally similar to those of the research-grade proteins, although the GMP-grade proteins tended to have greater proportions of complex glycans (Fig. 7). Analysis of the proteins from the second lot did not reveal any major differences, compared with the first lot (data not shown).

The oligomannose glycans were not equally distributed among the PNGSs. N262, N289/N295, N332, and N363 were enriched in oligomannose, corresponding to the intrinsic mannose patch that was noted previously for HIV Env. While present on all four proteins, the patch was more pronounced for clade A, B, and C proteins, while oligomannose was more evenly distributed on the clade AE protein. Clade C protein had an unusual enrichment in oligomannose at the C terminus (N406, N442, N448, and N463) that was absent in the other three proteins.

### Antigenicity analysis of the four gp120 proteins.

Finally, we sought to test whether any observed lot-to-lot variations in PNGS occupancy and glycan composition, no matter how minor they might be, had an effect on key antigenic features of these proteins. Using the affinity-measuring Octet QK^e^ system and a panel of probing reagents, we tested the preservation of key epitopes on the four gp120 proteins, including the CD4 binding site (CD4bs) (IgG-CD4 and MAb VRC01), V2 loop (MAb 2158), V3 region (MAb R16), gp120 bridging sheet and loop F overlapping the CD4bs (MAb R53), and glycan forms (MAb 2G12 and MAb PGT128). The epitope for the 2G12 antibody is thought to include mannose-rich glycans at positions 295, 332, 392, 386, and 448 (17). The PGT128 epitope includes glycans at positions 332 and 301, as well as the C-terminal end of the V3 loop (18). While we observed differences in affinities of these reagents, the differences between the two lots of each clade were minimal (Table 1); this included similar affinities of glycan-dependent 2G12 and PGT128 for lot 2 of the clade C protein, which exhibited the unusually large proportion of paucimannose, compared to lot 1. Thus, our results demonstrate that observed differences in glycan profiles did not result in changes in the affinity of antibodies targeting key epitopes of four gp120 proteins, including glycan-binding antibodies, indicating that proteins produced under different conditions mostly retained their antigenic structure.

**TABLE 1:** Binding affinity of two GMP lots of gp120 proteins

GMP gp120 protein and lot	*K_d_* (M)						
	MAb VRC01 (CD4bs)	MAb 2G12 (glycan)	MAb PGT128 (glycan)	MAb 2158 (V2)	MAb R16 (V3)	MAb R53 (C4)	IgG-CD4 (CD4bs)
Clade A, lot 1	6.04E−09	3.11E−10	7.68E−10	6.85E−09	5.59E−10	6.76E−08	5.48E−09
Clade A, lot 2	5.96E−09	1.80E−10	7.62E−10	6.46E−09	2.81E−10	2.76E−08	3.62E−09
Clade B, lot 1	3.11E−08	<1.0E−12	2.46E−09	4.55E−09	3.49E−10	1.16E−08	7.44E−09
Clade B, lot 2	2.83E−08	<1.0E−12	2.10E−09	3.26E−09	4.56E−10	1.32E−08	4.87E−09
Clade C, lot 1	4.53E−08	5.54E−09	3.25E−08	2.61E−08	3.80E−10	5.63E−09	1.57E−07
Clade C, lot 2	5.21E−08	2.24E−09	2.03E−08	3.31E−08	4.27E−10	4.98E−08	1.15E−07
Clade AE, lot 1	5.25E−08	8.02E−09	3.71E−08	3.05E−08	5.87E−10	8.89E−09	2.46E−08
Clade AE, lot 2	3.53E−08	8.62E−09	2.62E−08	3.32E−08	3.22E−10	5.63E−09	1.84E−08

## DISCUSSION

This report presents the first extensive comparison of glycosylation patterns of recombinant gp120 proteins from four clades of HIV-1 in two different cell lines, grown either at laboratory scale or under 50-liter GMP conditions, purified using different methods, and in two GMP lots prepared under identical conditions. Our results show that, in all four gp120 proteins included in the current study, the majority of PNGSs were occupied by glycans, with occupancy rates usually above 50%. We also found that, among glycans found on these proteins, oligomannose forms represented 40 to 50% and were concentrated in the previously described intrinsic mannose patch. Glycosylation profiles were basically very similar, with low levels of variability under different conditions. These differences did not affect binding by a panel of antibodies targeting key immunological epitopes of gp120 proteins, indicating that the observed low-level glycan differences should not have a major impact on protein immunogenicity.

The most prominent feature of HIV Env glycans distinguishing them from glycans on host proteins is the presence of oligomannose. In agreement with previous reports, we observed large proportions of oligomannose glycans (Man_5_ to Man_9_) on all four proteins produced under all conditions, confirming that this is a characteristic feature of HIV Env. Although previous studies reported a rather wide range of proportions of oligomannose, we consistently observed that it constituted 40 to 50% of glycans in all cases. A recent study reported that gp120 derived from infectious virions contained 50% oligomannose glycans (19), which suggests that glycosylation patterns of native Env are generally preserved in the recombinant gp120 proteins. This preservation is important for HIV vaccine development, aiming to elicit antibodies that bind and neutralize HIV virions.

The research-grade gp120 proteins were produced in 293F and CHO cells and purified using lectin columns, while the GMP-grade proteins were produced in CHO cells grown at 50-liter scale and purified using ion-exchange columns. However, we found only minor differences in glycan occupancy and glycan contents among proteins produced under different conditions, indicating that these features are primarily determined by viral sequence and not by the producing cells or purification process.

Our comparison of two independent GMP-grade lots of gp120 proteins showed consistent glycosylation patterns but also showed some differences, including a significant increase in paucimannose content in one of the lots of clade C protein. It should be noted that this particular lot differed from all other GMP lots in having a significantly higher yield of the protein. We do not have enough data to establish a causal relationship between these two observations, but we hypothesize that high levels of protein production overwhelmed medial and *trans*-Golgi glycan-processing machinery in the producing cells, resulting in secretion of proteins with glycans that were fully trimmed by *cis*-Golgi mannosidases but were incompletely branched, extended, and capped. Further work is needed to test the factors affecting the variability of glycosylation during GMP manufacturing, and acceptable variability levels should be established. Special attention should be paid to optimizations that boost protein production in cells, because that may have an impact on the cellular glycosylation machinery.

## MATERIALS AND METHODS

### Production of non-GMP gp120 proteins.

The four gp120 glycoproteins used in this report are from HIV-1 clade A isolate 92UG037.8, clade B isolate JRFL, clade C isolate 93MW965.26, and clade AE consensus (16). The non-GMP research-grade HIV-1 gp120 proteins were produced using two protein expression systems, i.e., a transiently transfected 293F cell expression system and a stably transfected CHO cell expression system. Codon-optimized gp120-coding DNA inserts cloned in the vector pJW4303 were used in both 293F and CHO cells. To produce gp120 proteins from transiently transfected 293F cells, the serum-free 293F cell supernatant was collected 72 h after transfection. To express gp120 proteins from CHO cells, the serum-free culture supernatant of stably transfected CHO cells was collected. The harvested research-grade gp120 proteins from both 293F and CHO cells were purified using a lectin column and verified by sodium dodecyl sulfate-polyacrylamide gel electrophoresis (SDS-PAGE) and Western blot analysis, as described previously (20–22).

### Production of GMP-grade gp120 proteins.

The codon-optimized gp120 gene inserts for the same four clades (A, B, C, and AE) as described above for research-grade proteins were transfected into CHO DG44 cells (Invitrogen, CA) and used to establish the master cell banks. CHO GD44 cells stably expressing each of the four gp120 were grown in 50-liter bioreactors, and the cell culture supernatants were collected after 8 to 10 days of fermentation and purified through a downstream purification process including anion-exchange, cation-exchange, and size exclusion steps, under GMP conditions. The purity of each gp120 protein was in the range of 96 to 98%, based on the release certificates. The same purified gp120 proteins are currently being tested in a phase I clinical trial (HVTN124) at six major U.S. medical centers.

### Detection of occupancy of PNGSs on gp120 proteins.

An aliquot of each gp120 protein was buffered to alkaline pH, reduced, alkylated, and digested with a combination of proteases, including Lys-C (Promega), Arg-C (Promega), Glu-C (Promega), and trypsin (Promega). Following digestion, the proteins were deglycosylated by endo H (Promega) and then PNGase F (Glyko; Prozyme) treatment in the presence of ^18^O-labeled water. The resulting peptides were separated on an Acclaim PepMap RSLC C_18_ column (75 μm by 15 cm) and eluted into the nanoelectrospray ion source of an Orbitrap Fusion Lumos Tribrid mass spectrometer (Thermo Fisher Scientific) with a 240-min linear gradient consisting of a 60-min wash in 100% solvent A followed by 0.8 to 80% acetonitrile over 180 min at a flow rate of 200 nl/min. Full MS analysis was conducted in the Orbitrap, and automated tandem mass spectrometry (MS/MS) analysis using collision-induced dissociation (CID) was conducted in the ion trap. The resulting data were analyzed using a combination of Proteome Discoverer (SEQUEST algorithm) and ProteoIQ (ProValT algorithm), to generate a 1% false-discovery rate for protein assignments. Site occupancy was calculated using spectral counts assigned to the ^18^O-Asp-containing (PNGase F-cleaved) and/or *N*-acetylhexosamine-modified (endo H-cleaved) peptides and their unmodified counterparts. The positivity cutoff value for spectral counts was set at 10% of the spectral count for the most abundant peptide in each LC-MS run. Peptides with spectral counts below the positivity cutoff value were not included in the analysis.

### *N*-linked glycan profiling analysis of gp120 proteins.

A 20-μg aliquot of each gp120 sample was denatured by boiling in SDS. Upon cooling, the SDS was removed by precipitation as its potassium salt. Denatured proteins were buffered, recombinant PNGase F was added, and the mixture was incubated overnight to release *N*-linked glycans. The released *N*-linked glycans were freed from residual enzyme, deglycosylated protein, and other contaminants by passage over a C_18_ Sep-Pak cartridge. The released purified glycans were permethylated using methyl iodide (CH_3_I) under basic conditions in an aprotic solvent (dimethyl sulfoxide), followed by recovery through organic extraction. For MS analysis, one-half of the total permethylated glycans released from 20 μg of protein (10 μg equivalent of protein) was supplemented by the addition of 10 pmol of an exogenous glycan standard (maltotetraose) that had been previously permethylated with isotopically heavy methyl iodide (^13^CH_3_I). The sample glycans spiked with standard were directly infused into an LTQ Orbitrap mass spectrometer fitted with an NSI interface (Orbitrap Discovery; Thermo-Fisher). Glycans were detected in full MS mode and by total ion mapping, in which automated CID is performed on small overlapping *m/z* windows. Total ion mapping allows the unbiased detection of ions that give fragmentation patterns consistent with glycan structural topologies (23). Glycan signal intensities were recovered from extracted full MS spectra (Xtract; Thermo-Fisher), and glycan identities were assigned based on the exact mass and CID fragmentation. Graphical representations of monosaccharide residues are presented in accordance with the broadly accepted symbolic nomenclature for glycans (SNFG) guidelines, and glycan analysis was performed in keeping with the minimum information required for a glycomics experiment (MIRAGE) guidelines for glycomic studies (24, 25).

### IgG-CD4 protein and gp120-specific MAbs.

The IgG-CD4 protein used in the Octet QK^e^ assays was a fusion protein of human CD4 domains 1 and 2 with human IgG1 Fc at the C terminus, produced by transient transfection of 293F cells and His-tagged purification. The gp120 CD4bs-specific MAb VRC01 (26) was produced from transiently transfected 293F cells using molecular clones coding for VRC01 heavy and light chains (obtained from the NIH AIDS Reagent Program) and was purified with a protein A column. The gp120 glycan-specific human MAb 2G12 (27) was purchased from Polymun Scientific. The gp120 glycan-specific MAb PGT128 (18) was provided by Wayne Koff from the International AIDS Vaccine Initiative. The gp120 V2-specific human MAb 2158 (28) was purchased from Susan Zolla-Pazner’s laboratory at Mount Sinai School of Medicine. The gp120 V3- and C4-specific rabbit MAbs R16 and R53 (29, 30) were produced from transiently transfected 293F cells using paired heavy and light chain molecular clones and were purified using a protein A column. The IgG-CD4 protein and MAbs produced in this study were verified before use.

### Antigenicity analysis of gp120 proteins with the Octet QK^e^ system.

The antigenicity of gp120 proteins was tested using IgG-CD4 and gp120-specific MAbs with the Octet QK^e^ system (ForteBio), based on biolayer interferometry. IgG-CD4 and each gp120-specific human MAb was individually loaded onto protein G sensors at 20 μg/ml, and the individual gp120-specific rabbit MAb was loaded onto protein A sensors at 10 μg/ml (diluted in ForteBio kinetics buffer). After capture, tips were washed in kinetics buffer, and a baseline measurement was recorded. The tips were then incubated in wells containing serial dilutions of individual gp120 protein (600 nM to 0.4 nM) to measure the association rate (*k*_on_) and dissociation rate (*k*_off_) constants. The antibody binding kinetics and *K_d_* values (*k*_off_/*k*_on_) were determined by the ForteBio data analysis software package v7.1, using a 1:1 fitting model for IgG-CD4 and MAbs VRC01, PGT128, 2G12, 2158, and R16 and using a 2:1 fitting model for MAb R53.

## References

[1] Reitter JN, Means RE, Desrosiers RC 1998 A role for carbohydrates in immune evasion in AIDS. Nat Med 4:679–684. doi:10.1038/nm0698-679. 9623976

[2] Mouquet H, Scharf L, Euler Z, Liu Y, Eden C, Scheid JF, Halper-Stromberg A, Gnanapragasam PNP, Spencer DIR, Seaman MS, Schuitemaker H, Feizi T, Nussenzweig MC, Bjorkman PJ 2012 Complex-type *N*-glycan recognition by potent broadly neutralizing HIV antibodies. Proc Natl Acad Sci U S A 109:E3268–E3277. doi:10.1073/pnas.1217207109. 23115339 PMC3511153

[3] Walker LM, Phogat SK, Chan-Hui P-Y, Wagner D, Phung P, Goss JL, Wrin T, Simek MD, Fling S, Mitcham JL, Lehrman JK, Priddy FH, Olsen OA, Frey SM, Hammond PW, Protocol G Principal Investigators, Kaminsky S, Zamb T, Moyle M, Koff WC, Poignard P, Burton DR 2009 Broad and potent neutralizing antibodies from an African donor reveal a new HIV-1 vaccine target. Science 326:285–289. doi:10.1126/science.1178746. 19729618 PMC3335270

[4] Bonomelli C, Doores KJ, Dunlop DC, Thaney V, Dwek RA, Burton DR, Crispin M, Scanlan CN 2011 The glycan shield of HIV is predominantly oligomannose independently of production system or viral clade. PLoS One 6:e23521. doi:10.1371/journal.pone.0023521. 21858152 PMC3156772

[5] Doores KJ, Bonomelli C, Harvey DJ, Vasiljevic S, Dwek RA, Burton DR, Crispin M, Scanlan CN 2010 Envelope glycans of immunodeficiency virions are almost entirely oligomannose antigens. Proc Natl Acad Sci U S A 107:13800–13805. doi:10.1073/pnas.1006498107. 20643940 PMC2922250

[6] Pritchard LK, Spencer DIR, Royle L, Bonomelli C, Seabright GE, Behrens A-J, Kulp DW, Menis S, Krumm SA, Dunlop DC, Crispin DJ, Bowden TA, Scanlan CN, Ward AB, Schief WR, Doores KJ, Crispin M 2015 Glycan clustering stabilizes the mannose patch of HIV-1 and preserves vulnerability to broadly neutralizing antibodies. Nat Commun 6:7479. doi:10.1038/ncomms8479. 26105115 PMC4500839

[7] Stanley P, Taniguchi N, Aebi M 2015 *N*-Glycans In Varki A, Cummings RD, Esko JD, Stanley P, Hart GW, Aebi M, Darvill AG, Kinoshita T, Packer NH, Prestegard JH, Schnaar RL, Seeberger PH (ed), Essentials of glycobiology, 3rd ed Cold Spring Harbor Laboratory Press, Cold Spring Harbor, NY. 27010055

[8] Go EP, Irungu J, Zhang Y, Dalpathado DS, Liao H-X, Sutherland LL, Alam SM, Haynes BF, Desaire H 2008 Glycosylation site-specific analysis of HIV envelope proteins (JR-FL and CON-S) reveals major differences in glycosylation site occupancy, glycoform profiles, and antigenic epitopes’ accessibility. J Proteome Res 7:1660–1674. doi:10.1021/pr7006957. 18330979 PMC3658474

[9] Go EP, Chang Q, Liao H-X, Sutherland LL, Alam SM, Haynes BF, Desaire H 2009 Glycosylation site-specific analysis of clade C HIV-1 envelope proteins. J Proteome Res 8:4231–4242. doi:10.1021/pr9002728. 19610667 PMC2756219

[10] Go EP, Herschhorn A, Gu C, Castillo-Menendez L, Zhang S, Mao Y, Chen H, Ding H, Wakefield JK, Hua D, Liao H-X, Kappes JC, Sodroski J, Desaire H 2015 Comparative analysis of the glycosylation profiles of membrane-anchored HIV-1 envelope glycoprotein trimers and soluble gp140. J Virol 89:8245–8257. doi:10.1128/JVI.00628-15. 26018173 PMC4524223

[11] Go EP, Liao H-X, Alam SM, Hua D, Haynes BF, Desaire H 2013 Characterization of host-cell line specific glycosylation profiles of early transmitted/founder HIV-1 gp120 envelope proteins. J Proteome Res 12:1223–1234. doi:10.1021/pr300870t. 23339644 PMC3674872

[12] Pritchard LK, Vasiljevic S, Ozorowski G, Seabright GE, Cupo A, Ringe R, Kim HJ, Sanders RW, Doores KJ, Burton DR, Wilson IA, Ward AB, Moore JP, Crispin M 2015 Structural constraints determine the glycosylation of HIV-1 envelope trimers. Cell Rep 11:1604–1613. doi:10.1016/j.celrep.2015.05.017. 26051934 PMC4555872

[13] Rerks-Ngarm S, MOPH-TAVEG Investigators, Pitisuttithum P, Nitayaphan S, Kaewkungwal J, Chiu J, Paris R, Premsri N, Namwat C, de Souza M, Adams E, Benenson M, Gurunathan S, Tartaglia J, McNeil JG, Francis DP, Stablein D, Birx DL, Chunsuttiwat S, Khamboonruang C, Thongcharoen P, Robb ML, Michael NL, Kunasol P, Kim JH 2009 Vaccination with ALVAC and AIDSVAX to prevent HIV-1 infection in Thailand. N Engl J Med 361:2209–2220. doi:10.1056/NEJMoa0908492. 19843557

[14] Wen Y, Trinh HV, Linton CE, Tani C, Norais N, Martinez-Guzman D, Ramesh P, Sun Y, Situ F, Karaca-Griffin S, Hamlin C, Onkar S, Tian S, Hilt S, Malyala P, Lodaya R, Li N, Otten G, Palladino G, Friedrich K, Aggarwal Y, LaBranche C, Duffy R, Shen X, Tomaras GD, Montefiori DC, Fulp W, Gottardo R, Burke B, Ulmer JB, Zolla-Pazner S, Liao H-X, Haynes BF, Michael NL, Kim JH, Rao M, O'Connell RJ, Carfi A, Barnett SW 2018 Generation and characterization of a bivalent protein boost for future clinical trials: HIV-1 subtypes CR01_AE and B gp120 antigens with a potent adjuvant. PLoS One 13:e0194266. doi:10.1371/journal.pone.0194266. 29698406 PMC5919662

[15] Zambonelli C, Dey AK, Hilt S, Stephenson S, Go EP, Clark DF, Wininger M, Labranche C, Montefiori D, Liao H-X, Swanstrom RI, Desaire H, Haynes BF, Carfi A, Barnett SW 2016 Generation and characterization of a bivalent HIV-1 subtype C gp120 protein boost for proof-of-concept HIV vaccine efficacy trials in southern Africa. PLoS One 11:e0157391. doi:10.1371/journal.pone.0157391. 27442017 PMC4956256

[16] Wang S, Chou T-H, Hackett A, Efros V, Wang Y, Han D, Wallace A, Chen Y, Hu G, Liu S, Clapham P, Arthos J, Montefiori D, Lu S 2017 Screening of primary gp120 immunogens to formulate the next generation polyvalent DNA prime-protein boost HIV-1 vaccines. Hum Vaccin Immunother 13:2996–3009. doi:10.1080/21645515.2017.1380137. 28933684 PMC5718816

[17] Sanders RW, Venturi M, Schiffner L, Kalyanaraman R, Katinger H, Lloyd KO, Kwong PD, Moore JP 2002 The mannose-dependent epitope for neutralizing antibody 2G12 on human immunodeficiency virus type 1 glycoprotein gp120. J Virol 76:7293–7305. doi:10.1128/jvi.76.14.7293-7305.2002. 12072528 PMC136300

[18] Pejchal R, Doores KJ, Walker LM, Khayat R, Huang P-S, Wang S-K, Stanfield RL, Julien J-P, Ramos A, Crispin M, Depetris R, Katpally U, Marozsan A, Cupo A, Maloveste S, Liu Y, McBride R, Ito Y, Sanders RW, Ogohara C, Paulson JC, Feizi T, Scanlan CN, Wong C-H, Moore JP, Olson WC, Ward AB, Poignard P, Schief WR, Burton DR, Wilson IA 2011 A potent and broad neutralizing antibody recognizes and penetrates the HIV glycan shield. Science 334:1097–1103. doi:10.1126/science.1213256. 21998254 PMC3280215

[19] Struwe WB, Chertova E, Allen JD, Seabright GE, Watanabe Y, Harvey DJ, Medina-Ramirez M, Roser JD, Smith R, Westcott D, Keele BF, Bess JW, Sanders RW, Lifson JD, Moore JP, Crispin M 2018 Site-specific glycosylation of virion-derived HIV-1 Env is mimicked by a soluble trimeric immunogen. Cell Rep 24:1958–1966.e5. doi:10.1016/j.celrep.2018.07.080. 30134158 PMC6113929

[20] Vaine M, Wang S, Hackett A, Arthos J, Lu S 2010 Antibody responses elicited through homologous or heterologous prime-boost DNA and protein vaccinations differ in functional activity and avidity. Vaccine 28:2999–3007. doi:10.1016/j.vaccine.2010.02.006. 20170767 PMC2847033

[21] Wang S, Pal R, Mascola JR, Chou T-H, Mboudjeka I, Shen S, Liu Q, Whitney S, Keen T, Nair BC, Kalyanaraman VS, Markham P, Lu S 2006 Polyvalent HIV-1 Env vaccine formulations delivered by the DNA priming plus protein boosting approach are effective in generating neutralizing antibodies against primary human immunodeficiency virus type 1 isolates from subtypes A, B, C, D and E. Virology 350:34–47. doi:10.1016/j.virol.2006.02.032. 16616287

[22] Wang S, Kishko M, Wan S, Wang Y, Brewster F, Gray GE, Violari A, Sullivan JL, Somasundaran M, Luzuriaga K, Lu S 2012 Pilot study on the immunogenicity of paired Env immunogens from mother-to-child transmitted HIV-1 isolates. Hum Vaccin Immunother 8:1638–1647. doi:10.4161/hv.22414. 23151449 PMC3601138

[23] Aoki K, Perlman M, Lim J-M, Cantu R, Wells L, Tiemeyer M 2007 Dynamic developmental elaboration of *N*-linked glycan complexity in the *Drosophila melanogaster* embryo. J Biol Chem 282:9127–9142. doi:10.1074/jbc.M606711200. 17264077

[24] Varki A, Cummings RD, Aebi M, Packer NH, Seeberger PH, Esko JD, Stanley P, Hart G, Darvill A, Kinoshita T, Prestegard JJ, Schnaar RL, Freeze HH, Marth JD, Bertozzi CR, Etzler ME, Frank M, Vliegenthart JF, Lütteke T, Perez S, Bolton E, Rudd P, Paulson J, Kanehisa M, Toukach P, Aoki-Kinoshita KF, Dell A, Narimatsu H, York W, Taniguchi N, Kornfeld S 2015 Symbol nomenclature for graphical representations of glycans. Glycobiology 25:1323–1324. doi:10.1093/glycob/cwv091. 26543186 PMC4643639

[25] York WS, Agravat S, Aoki-Kinoshita KF, McBride R, Campbell MP, Costello CE, Dell A, Feizi T, Haslam SM, Karlsson N, Khoo K-H, Kolarich D, Liu Y, Novotny M, Packer NH, Paulson JC, Rapp E, Ranzinger R, Rudd PM, Smith DF, Struwe WB, Tiemeyer M, Wells L, Zaia J, Kettner C 2014 MIRAGE: the minimum information required for a glycomics experiment. Glycobiology 24:402–406. doi:10.1093/glycob/cwu018. 24653214 PMC3976285

[26] Wu X, Yang Z-Y, Li Y, Hogerkorp C-M, Schief WR, Seaman MS, Zhou T, Schmidt SD, Wu L, Xu L, Longo NS, McKee K, O'Dell S, Louder MK, Wycuff DL, Feng Y, Nason M, Doria-Rose N, Connors M, Kwong PD, Roederer M, Wyatt RT, Nabel GJ, Mascola JR 2010 Rational design of envelope identifies broadly neutralizing human monoclonal antibodies to HIV-1. Science 329:856–861. doi:10.1126/science.1187659. 20616233 PMC2965066

[27] Trkola A, Purtscher M, Muster T, Ballaun C, Buchacher A, Sullivan N, Srinivasan K, Sodroski J, Moore JP, Katinger H 1996 Human monoclonal antibody 2G12 defines a distinctive neutralization epitope on the gp120 glycoprotein of human immunodeficiency virus type 1. J Virol 70:1100–1108. doi:10.1128/JVI.70.2.1100-1108.1996. 8551569 PMC189917

[28] Pinter A, Honnen WJ, He Y, Gorny MK, Zolla-Pazner S, Kayman SC 2004 The V1/V2 domain of gp120 is a global regulator of the sensitivity of primary human immunodeficiency virus type 1 isolates to neutralization by antibodies commonly induced upon infection. J Virol 78:5205–5215. doi:10.1128/jvi.78.10.5205-5215.2004. 15113902 PMC400352

[29] Chen Y, Vaine M, Wallace A, Han D, Wan S, Seaman MS, Montefiori D, Wang S, Lu S 2013 A novel rabbit monoclonal antibody platform to dissect the diverse repertoire of antibody epitopes for HIV-1 Env immunogen design. J Virol 87:10232–10243. doi:10.1128/JVI.00837-13. 23864612 PMC3754024

[30] Pan R, Chen Y, Vaine M, Hu G, Wang S, Lu S, Kong X-P 2015 Structural analysis of a novel rabbit monoclonal antibody R53 targeting an epitope in HIV-1 gp120 C4 region critical for receptor and co-receptor binding. Emerg Microbes Infect 4:e44. doi:10.1038/emi.2015.44. 26251831 PMC4522616

